# Powder Filling and Sintering of 3D In-chip Solenoid Coils with High Aspect Ratio Structure

**DOI:** 10.3390/mi11030328

**Published:** 2020-03-22

**Authors:** Yujia Huang, Haiwang Li, Jiamian Sun, Yanxin Zhai, Hanqing Li, Tiantong Xu

**Affiliations:** 1 National Key Laboratory of Science and Technology on Aero Engines Aero-Thermodynamics, Beihang University, Beijing 100191, China; hyujia96@outlook.com (Y.H.); 09620@buaa.edu.cn (H.L.); sunjiamian@buaa.edu.cn (J.S.); Alanzhai@buaa.edu.cn (Y.Z.); 2 Microsystems Technology Laboratories, Massachusetts Institute of Technology, Boston, MA 02139, USA; hqli@mit.edu

**Keywords:** through-silicon-vias (TSVs), three-dimensional (3D) solenoid coils, microelectromechanical system (MEMS), powder filling, metal powder sintering

## Abstract

In this study, a 3D coil embedded in a silicon substrate including densely distributed through-silicon vias (TSVs) was fabricated via a rapid metal powder sintering process. The filling and sintering methods for microdevices were evaluated, and the effects of powder types were compared. The parameters influencing the properties and processing speed were analyzed. The results showed that the pre-alloyed powder exhibited the best uniformity and stability when the experiment used two or more types of powders to avoid the segregation effect. The smaller the particle diameter, the better the inductive performance will be. The entire structure can be sintered near the melting point of the alloy, and increasing the temperature increases strength, while resulting in low resistivity. Finally, an 800-µm-high coil was fabricated. This process does not need surface metallization and seed layer formation. The forming process involves only sintering instead of slowly growing copper with a tiny current. Therefore, this process has advantages, such as a process time of 7 h, corresponding to an 84% reduction compared to current electroplating processes (45 h), and a 543% efficiency improvement. Thus, this process is more efficient, controllable, stable, and suitable for mass production of devices with flexible dimensions.

## 1. Introduction

With the miniaturization of electronic devices, increasing attention has been focused on microelectromechanical system (MEMS) devices [1,2,3]. Meanwhile, semiconductor technology increasingly demands feature size reduction and efficiency improvements. Three-dimensional (3D) integration technology has become a promising method to improve the performance of integrated circuits. The realization of electrical interconnection of multistacked chips has become the research focus, which can help reduce the size of MEMS devices and improve power density [4,5,6]. Three-dimensional microcoils that are embedded in silicon substrates via high-aspect-ratio structure etching can achieve shorter interconnections in the vertical direction. This technology makes full use of the vertical space in the chips to obtain higher power density and improve performance significantly [7].

The usual process for constructing a 3D coil in a silicon substrate is copper electroplating. Copper electroplating is extensively used in the fabrication of structures with through-silicon-vias (TSVs) or achieving interconnections between wafers, owing to their low resistivity and higher resistance to electromigration, which has been reported in many studies, and has become a relatively mature method in standard MEMS processes. However, this process invariably takes a long time to fill the deep holes from the bottom to the top. Li et al. [8] reported that electroplating of 1000 µm-deep TSVs with a diameter of 100 µm was completed in 45 h; this is the most successful fabrication of a 3D coil using the deepest and smallest TSVs. It also requires seed layer deposition before plating and accurate surface planarization after plating. Moreover, for a 3D structure formed using two or more wafers bonded together, this wet process could cause liquid to penetrate any void defects between the wafers and other small gaps and holes, which can significantly damage the performance of microdevices.

To overcome these disadvantages and achieve rapid prototyping, this work employs the dry metal powder filling and sintering method, which is commonly used in large-scale applications for microfabricated silicon wafers comprising micrometal particles. Powder sintering refers to the process of using metal powders or powder compacts to obtain the required strength and characteristics of materials or products. The basic principles are physical and chemical actions such as bonding between metal particles occurring when the powder is heated to a temperature lower than the melting point of the main component.

Copper and its alloys have been widely used in large-scale sintering processes because of their good thermal and electrical properties. Ja-myeong Koo et al. [9] added copper and iron oxide powders to iron powder during sintering to improve charge performance. Pyo-Hyun et al. [10] reported that a copper-plated graphite/copper composite significantly enhanced the sinterability of brushes during direct current (DC) motor fabrication. Seijiro Maki et al. [11] sintered copper powder mixed with graphite powder to study the resistance sintering characteristics of these electrolytic powders. Furthermore, Gang Qin et al. [12] performed laser sintering of a copper paste with fine particles to generate electrically conductive copper films with a low resistivity of 1.05 × 10^−5^ Ω·cm. The main features of the studies mentioned above are that the sintered structures are simple and mostly linear or planar; moreover, all the processes are carried out on a large scale. Furthermore, there is no relevant research about using sintering methods in micron rank to fabricate 3D complex microstructures because of the difficulties in filling and sintering. However, with the increasing popularity of microdevices, innovations are necessary in microscale metal-forming technology (electroplating, magnetron sputtering, etc.). 

Hence, in this study, the optimized conventional sintering process was adjusted and combined with the MEMS process for fabricating 3D complex microstructures. A highly conductive coil was ultimately obtained; this process is a novel method for achieving electrical connections and fabricating an integrated circuit (IC)/MEMS-compatible coil. This method can fundamentally prevent the possible adverse effects of a liquid environment. In addition, it will significantly improve production efficiency without the need for complex processes such as surface metallization and seed layer formation.

## 2. Design and Fabrication 

**Figure 1:**
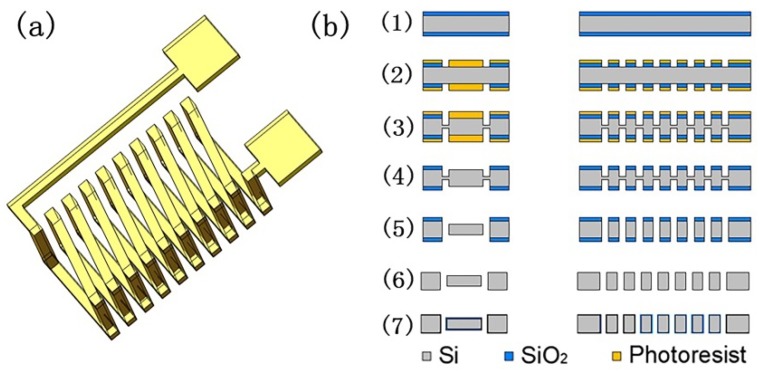
(**a**) 3D schematic of an in-chip solenoid metal wiring and (**b**) fabrication procedures of the 3D in-chip solenoid cavities.

An 800 μm-thick monocrystalline silicon wafer with a 2 μm-thick oxidation layer on both sides was used in this research. In previous studies, we compared the experimental results of 500, 800, and 1000 µm-thick silicon wafers, and the results showed that the change in thickness did not affect the sintering process parameters (such as the sintering time and vibration conditions). Therefore, this experiment was performed using an 800 μm-thick silicon wafer; the sintering parameters (powder type, sintering temperature, and particle size) were changed to achieve microscale metal sintering. The 800 µm high in-chip solenoid cavities composed of vertical parts with cross sections of 300 µm × 300 µm and horizontal parts with cross sections of 300 µm × 200 µm were fabricated first. The fabrication process is depicted in Figure 1. (1) The wafer surface was cleaned using the piranha solution (98% H_2_SO_4_: 30% H_2_O_2_ = 3:1) before coating the photoresist (AZ4620, AZ Electronic Material, Suzhou, China). (2) The coil pattern with the mask was exposed and then the oxide layer on the exposed area was removed via the buffered oxide etch (BOE) dip process. (3) The photoresist was used as a mask layer to etch horizontal trench patterns to a depth of 150 μm. (4) The remaining photoresist layer was removed using an acetone solution. (5) An oxide layer was used as another mask layer to etch through-hole patterns to depths of 400 μm; meanwhile, the horizontal trench patterns without the photoresist over them were etched to depths of 200 μm. (6) All SiO_2_ layers were removed using a diluted HF solution. (7) Thermal oxidation was used to grow a thick SiO_2_ layer as an isolating layer between turns. In steps (3) and (5) of this experiment, the etching process was finished using an inductively coupled plasma (ICP) (SPTS Company, Newport, UK). Etching must be conducted in a low-temperature environment in which helium is supplied to the backside of the wafer as the cooling gas. The whole etching process was divided into two steps: an etching step with sulfur hexafluoride gas (SF_6_) and a passivation step with octafluoroyclobutane gas (C_4_F_8_). The entire etching process was divided into two steps because the coil involved a high-aspect-ratio microstructure. Once one side was etched to the set depth, the silicon wafer was flipped and the same structure was etched on the opposite side. After the structure of the solenoid cavities was obtained, the entire wafer was cut into dies to facilitate the subsequent sintering process. 

**Figure 2:**
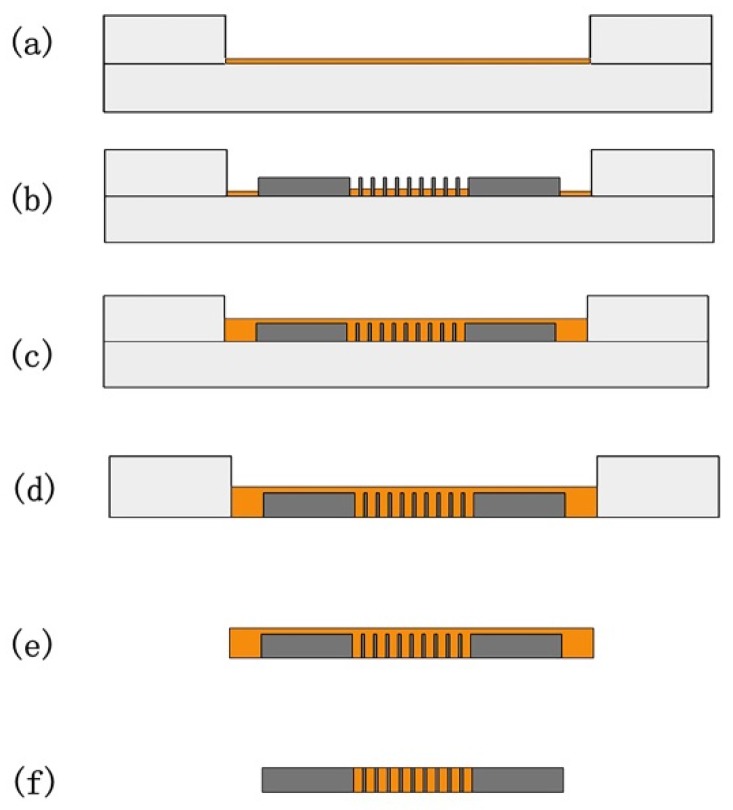
3D coil manufacturing steps with double-sided filling method: (**a**) fix the trenched quartz glass plate to the bottom plate and pour a layer of powder; (**b**) place the dies with solenoid cavities over the powder; (**c**) continue pouring the powder until it completely covers the silicon structure; (**d**) after vibrating and sintering, separate the upper and lower quartz glass plates; (**e**) remove the sample; (**f**) a sintered coil can be obtained after surface lapping cleaning.

After fabricating the solenoid cavities inside the substrate, the next step was a powder filling and sintering process, which is shown in Figure 2. The assembly was placed on a vibrating table and shaken at a set frequency for 120 s. Studies have reported that when the filling material is a spherical powder with particle sizes in the order of 10–100 µm, it is appropriate to use a vibration frequency of 10–100 Hz [13,14]. At the same time, there are different optimal vibration frequencies for different kinds of powders and different working environments. The frequency range is mostly 20–60 Hz, so 40 Hz was selected as the frequency for this experiment. In addition, vibration in one direction is better than that in multiple directions [15]. Therefore, this study used vertical vibration. Investigation on the vibration time shows that it is generally less than 120 s, but after reaching a stable state, lengthening the vibration time has negligible effects on the filling process [16,17]; hence, a relatively long vibration time of 120 s was selected to achieve sufficient filling. Then, the sample was loaded in an annealing furnace and heated to a set temperature ranging from 600 °C to 800 °C under forming gas for 7 h. The whole process was divided into the heating stage (3 h), thermal insulation stage (1 h), and cooling stage (3 h). The heating and cooling time were related to the requirements of the annealing furnace process.

To ensure that sintering was performed in a reducing atmosphere to prevent oxidation of the powder, hydrogen as reducing gas needed to be continuously passed into the furnace during the whole process. At the same time, because of the explosion risk of hydrogen, it needed to be mixed with protective nitrogen in a certain proportion (nitrogen: hydrogen = 95:5). First, the furnace was vacuumed, and then the gas was introduced into the furnace to ensure micropositive pressure. This process was repeated two–three times. The gas flow at the outlet was adjusted while ensuring that the furnace was stable under micropositive pressure conditions. Then the heating process was carried out.

**Figure 3:**
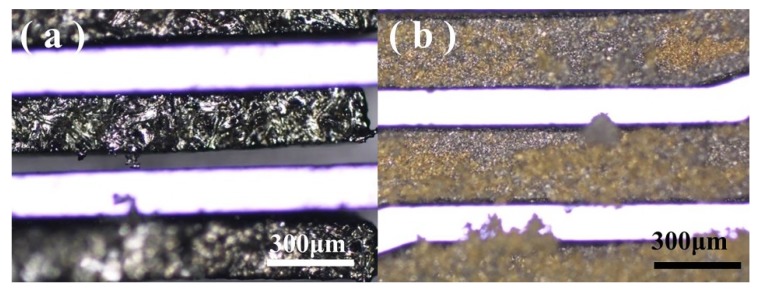
(**a**) After the solidification of tin, significant volume shrinkage occurred, and (**b**) a portion of the tin-rich region also underwent volumetric shrinkage after sintering the pre-mixed powder.

Table 1 lists the characteristics of the different types of powder used in this study. Tin was chosen because of its low melting temperature (230 °C) and its ability to combine with many metals to form various alloys. It is widely used in electronics, semiconductors, and other fields. The tin powder was mechanically milled to obtain a powder with spherical particles, because they aid in efficient packing when compared to irregular particle shapes and also enable better performance in powder filling [18]. Inhomogeneity caused by manual mixing led to the accumulation of tin in Figure 3b, which melted first during the experiment, and the volume shrinkage after solidification caused a significant volume change, as shown in Figure 3, which will affect electrical performance and physical strength.

**Figure 4:**
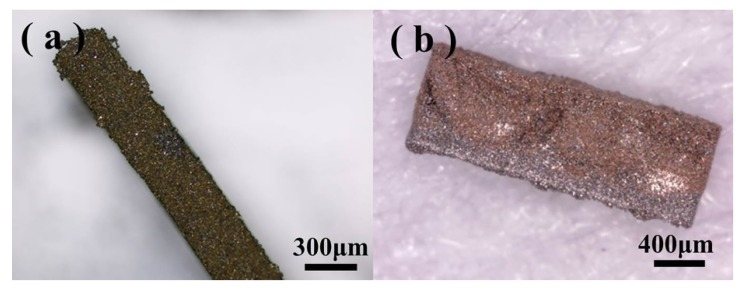
Sample after sintering of the 37 µm Cu powder mixed with 5 µm Sn powder in the through-trench. (**a**) Copper was observed to occupy most of the field on the upper surface and (**b**) a distinct stratification can be seen on the side.

Additionally, when there were two or more sintered powder types, obvious stratification occurred due to unavoidable segregation because of the Brazil nut problem (BNP), as shown in Figure 4. In the BNP, hard spheres with larger diameters rise to the top, which is a common type of segregation [19,20,21]. Besides this, the segregation exhibited various patterns, such as the reverse Brazil-nut effect (RBNE), sandwich pattern (SP), axial or radial streaks, and sliding spontaneous stratification [22].

There are many factors that cause the powder segregation effect: the properties of particles, such as size, shape, density, elasticity, and roughness; the environment of the particle bed, such as the shape of the vessel, the filling depth, the gap fluid, and the temperature and humidity. Moreover, there are also different mechanisms proposed for this phenomenon, including void filling, density-driven phenomena, inertia, buoyancy, convection, geometric shapes of particles, and the influence of different frictions and interstitial media [22]. Thus, the segregation effect has strong inevitability and uncertainty [23,24,25,26]. Therefore, pre-alloyed powder with a specific particle composition (M_Sn_ = 44.83% and M_Cu_ = 55.17%) was selected for this experiment, with a melting point of approximately 650 °C according to the copper–tin phase diagram [27]. Each particle in the pre-alloyed powder contains various metal elements of the alloy, which is more uniform than that obtained by mechanically mixing multiple single metal powders. In the sintering process, no segregation or melting of low-melting-point metals occurs first, so it is widely used in the powder metallurgy industry [28,29,30]. In addition to temperature, powder size is one of the parameters affecting sintering conditions in conventional sintering [31,32,33,34]. To verify whether diameters of the powders will affect the performance of the microstructures, two pre-alloyed powders with different diameters were selected.

It was necessary to ensure that the powder used in the experiment experiences no oxidation, because if oxidation occurs, the melting point will increase greatly.

## 3. Results and Discussion

**Figure 5:**
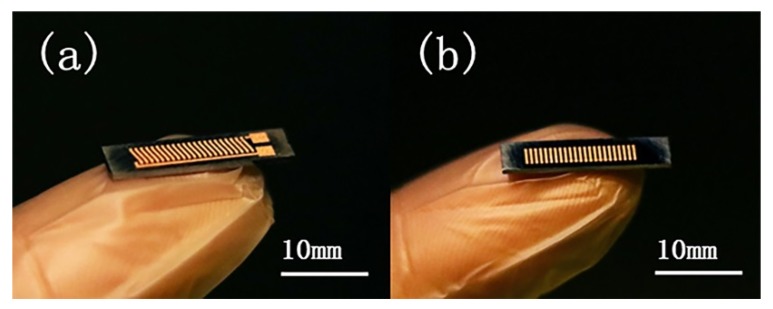
(**a**) Upper and (**b**) lower surfaces of the coil obtained by sintering the pre-alloyed powder after surface cleaning.

**Figure 6:**
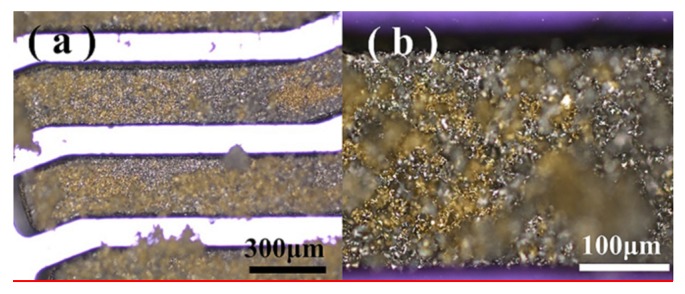
Upper surface of the sample obtained by sintering (650 °C) the pre-mixed powder (5 μm diameter copper and tin) in the trench structure with (**a**) 50× and (**b**) 200× magnifications.

**Figure 7:**
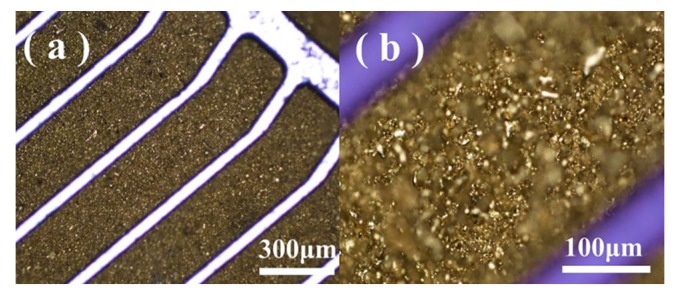
Upper surface of the sample obtained by sintering (650 °C) the 20 μm diameter pre-alloyed powder in the trench structure with (**a**) 50× and (**b**) 200× magnifications.

A complete in-chip 3D coil can be obtained after finishing the process flow. Figure 5 depicts the (**a**) upper and (**b**) lower surfaces of a Cu-Sn pre-alloyed powder-filled chip after surface lapping cleaning. Figure 6 shows the pre-mixed powder trench structure at different magnifications, and Figure 7 shows the trench filled with pre-alloyed powder. The filling condition of pre-alloyed powder was observed to be much more uniform, indicating that the sintering effect could be good. After removing the silicon structure, a 3D coil could ultimately be obtained.

**Figure 8:**
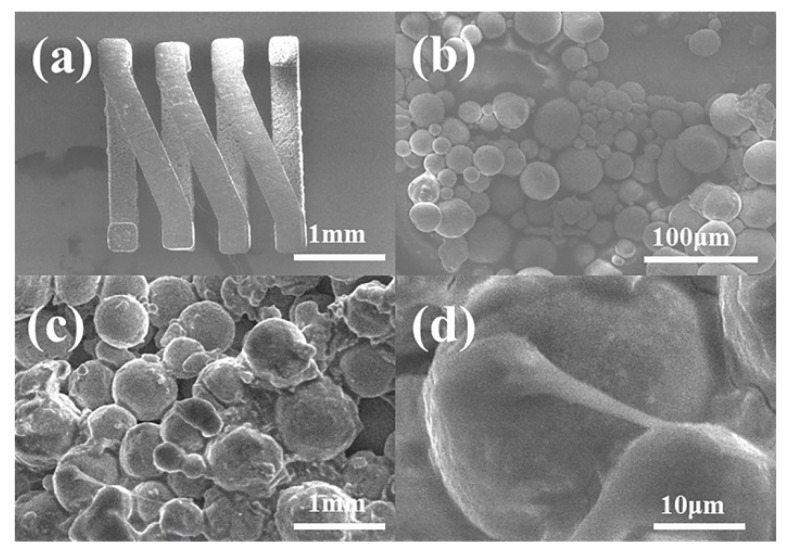
(**a**) Overall structure after removing the silicon substrate; (**b**) pre-alloyed powder before sintering; (**c**) after sintering; and (**d**) adhesion between particles after sintering.

Figure 8 presents scanning electron microscope (SEM) images of the pre-alloyed powder coil after removing the silicon substrate with tetramethylammonium hydroxide (TMAH) solution. Figure 8a shows the completely sintered structure of the coil after removing the silicon substrate, indicating that the coil has a certain physical strength. Figure 8c,d indicates that most of the powder particles remained spherical; however, there were obvious adhesions between the particles compared to that observed in Figure 8a, proving that part of the particles melted under the set temperature. The increasing temperature facilitated the interaction of the contact surfaces of the particles; furthermore, the contact surfaces gradually expanded and formed grain boundaries, resulting in bonding and rearrangement. However, a point-to-point contact still prevailed among the particles, and the total surface area was not observed to decrease. This is because when sintering large-sized structures, the process needs high pressure, but this is not suitable for producing 3D complex microstructures. The lack of a pressing process increases the sintered porosity to increase and decreases the density, thereby making it difficult to form a high-strength sintered structure. However, the coil can keep a good and complete shape on the silicon substrate. As the interaction between the particles increases, the resistivity decreases with an increase in strength.

Table 2 lists the resistivities of the sintered samples with different particle diameters under different sintering temperatures (600–800 °C). Because of the significant difference between the expansion rate of the alloy and silicon, the silicon structure will crack when the temperature is too high (900 °C). Therefore, the sintering temperature was not allowed to exceed 800 °C. 

**Figure 9:**
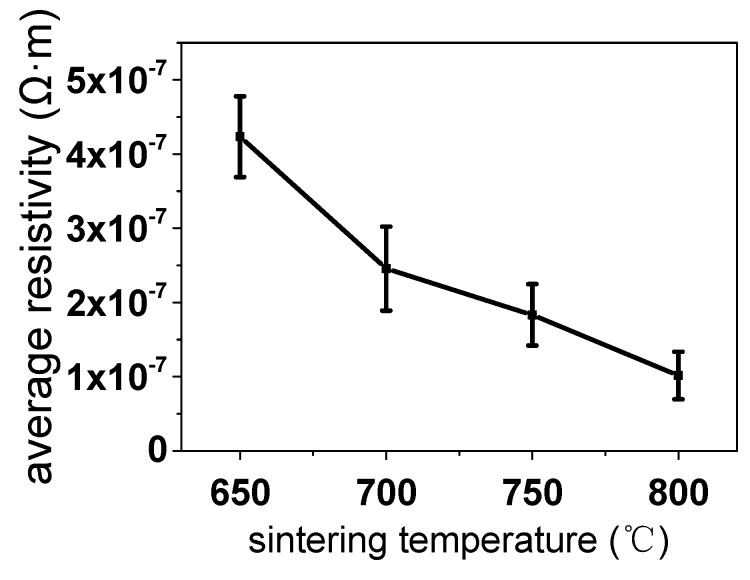
Resistivities obtained by sintering at different temperatures (600–800 °C).

Table 2 and Figure 9 show that the resistivity values exhibited a significant downward trend with increasing temperatures. On increasing the sintering temperature, diffusion and grain boundary diffusion occurred between the powder particles, metal connections were formed between particles, and the sintering neck began to grow. In the diffusion process, according to Fick’s first law of diffusion, the higher the temperature, the higher the diffusion coefficient and the more obvious the diffusion effect. After the formation of new grains, the connection between the particles changed from a weak connection involving Van der Waals forces to a strong connection involving crystal contact. According to the formula of the equilibrium concentration of vacancies in the crystal, the higher the temperature, the higher the concentration of vacancies; this promotes mass transfer and densification. Therefore, the interface between particles becomes smaller after sintering, crystal grains become larger, and the rate of free electron conduction increases.

Meanwhile, Table 2 shows that decreasing the particle diameter will decrease the resistivity significantly. This is because the smaller the radius, the smaller the porosity. At the same time, local amorphization is more obvious in larger particles, which will hinder the entire sintering process; the surface grain-boundary diffusion also requires a longer time or higher temperature for large particles. In addition, owing to agglomeration of powder particles, the adhesion between large particles appears uneven, indicating that the particles with smaller particle diameters have a higher degree of densification during the sintering process. The smaller the particle size, the more uniform the heat energy transfer between the particles, and the higher the overall heat absorption.

At 25 °C, the resistivity of the samples produced by this method (6.50 × 10^−8^ Ω·m) exceeded that of the samples produced via soldering (1.14 × 10^−7^ Ω·m), indium (8.37 × 10^−8^ Ω·m), and silver paste (8.67 × 10^−7^ Ω·m) produced under the same temperature [35].

## 4. Conclusions

In this study, a 3D microcoil embedded in a silicon substrate was fabricated via metal powder filling and sintering in silicon cavities etched using MEMS processes. Metal powders were filled in the 800 µm high silicon cavities and sintered for 7 h in forming gas to construct the 3D coils. Coils sintered using the pre-alloyed powder exhibited advantages such as good fluidity, low sintering temperature, and better sintering performance; it could fundamentally avoid segregation and stratification between particles. The minimum DC resistivity of the coil could reach 6.50 × 10^−8^ Ω·m. The conductivity could be improved considerably by reducing the diameter of the powder particles. Furthermore, the sintering temperature was observed to be inversely proportional to the resistivity. Meanwhile, higher temperatures also improved the strength and yield by increasing adhesion between particles. This batch fabrication method achieved similar resistivity values between batches and significantly simplified the metal filling process; this was achieved by eliminating the need for seed layer deposition, which reduced the processing time from days to hours. This method significantly improved process efficiency compared to electroplating, especially for meeting the increasing demands of TSV technology. The electroplating time showed an approximately linear increasing trend when through-hole structures became deeper, but the sintering time did not change with the structure depth. At the same time, the sintering process eliminated the influence of the wet environment on the fabricated device, thus making the process more stable and controllable. Considering these advantages, the process mentioned above gives a new option for metal filling in microdevices. According to the requirements and characteristics of MEMS standard processes, having choices such as electroplating and sintering can considerably increase the diversity of micromachining processes. 

However, compared to electroplating, the powder metal sintering method produces products with insufficient density and resistivity. Hence, to address these limitations, the use of higher temperatures and smaller powder diameters will be elucidated in future experiments to improve density and resistivity.

## Figures and Tables

**Table 1:** Types and properties of powders.

Powder Type	Volume Ratio of Cu to Sn	Average Particle Diameter (μm)	Melting Point (°C)	Density (g/cm³)
Pre-alloyed powder	1:1	20	600–700 (M_Sn_ = 44.83%)	8.120
Pre-alloyed powder	1:1	10	600–700 (M_Sn_ = 44.83%)	8.120
Pre-mixed powder	1:1	5	T_Sn_ = 231.89/T_Cu_ = 1083.4	8.120
Cu		37	1083.4	8.960
Cu		5	1083.4	8.960
Sn		5	231.89	7.280

**Table 2:** Properties of different sintering temperatures and diameters of samples.

Sample Number	Sintering Temperature (°C)	Average Particle Diameter (μm)	Resistivity (Ω·m^2^/m)
1	800	10	7.17 × 10^−8^
2	800	10	1.15 × 10^−7^
3	800	10	6.50 × 10^−8^
4	750	10	2.31 × 10^−7^
5	750	10	1.64 × 10^−7^
6	750	10	1.55 × 10^−7^
7	700	10	3.11 × 10^−7^
8	700	10	2.17 × 10^−7^
9	700	10	2.09 × 10^−7^
10	650	10	4.62 × 10^−7^
11	650	10	3.85 × 10^−7^
12	700	20	1.20 × 10^−5^
13	650	20	3.16 × 10^−5^
14	600	20	8.06 × 10^−6^
